# A Giant Pediatric Ossifying Fibroma: A Case Report of a Rare Occipital Presentation

**DOI:** 10.7759/cureus.101980

**Published:** 2026-01-21

**Authors:** Ramón Castruita Meza, Oswaldo M Soriano, Glaucia B Tola Tarqui, Samuel García Torres, Vicente González Carranza

**Affiliations:** 1 Pediatric Neurosurgery, Hospital Infantil de México Federico Gómez, Ciudad de México, MEX

**Keywords:** neuro oncology, occipital, ossifying fibroma, pediatric neuro surgery, pediatric tumors

## Abstract

Ossifying fibroma is a very uncommon tumor that affects craniofacial structures. Occipital bone presentation is extremely rare; it is more commonly found in the jaw. Its locally aggressive behavior and expansion near brain tissue require total surgical removal.

We report a nine-year-old girl with a five-year history of a growing, painless mass in the occipital region of the skull and progressive loss of vision. In the imaging studies, an extra-axial mass arising from the occipital bone was identified, with significant compression of the brain parenchyma. An occipital craniectomy and complete en bloc resection were performed, with histopathology confirming ossifying fibroma. Improvement in vision after surgery was noted. To our knowledge, this is the fifth reported case and is complemented by a review of the literature. The uncommon presentation of bone tumors, such as ossifying fibroma, with locally aggressive behavior should always be considered in the differential diagnosis in the pediatric population, because total resection decreases morbidity and can be curative.

## Introduction

Ossifying fibroma is a benign fibrous-osseous tumor within a group of lesions of the mandible and craniofacial bones that share the characteristic of replacing normal bone with fibrous tissue containing foci of mineralization [1]. Described in 1872 by Menzel [2], it comprises a clinical spectrum that depends on the location, is often complemented by imaging studies, and is corroborated by histopathology. 

Ossifying fibroma is characterized by being a progressively growing lesion with bone expansion. Microscopically, it contains bone tissue in a stroma of fibrous connective tissue. Its classification includes three entities: cemento-ossifying fibroma (of the jaw), juvenile trabecular ossifying fibroma, and psammomatous juvenile ossifying fibroma - all of them most commonly appearing in the jaw, maxillary sinus, and paranasal sinuses, and less commonly in the nasal cavity [3].

Lesions in the cranial bone region are extremely rare; there are no reliable statistics on their prevalence. Some case reports describe them as isolated lesions that may occur in the second or third decade of life, predominantly in females [2]. When found in the calvaria, they generally present with symptoms of bone expansion and pain and may mimic a malignant neoplasm due to rapid and aggressive growth. Neurological symptoms may be associated with compression of intracranial structures [2].

To date, very few documented cases exist in both adult and pediatric populations regarding the atypical presentation of this type of lesion in the cranial vault. This case aims to contribute to documenting the diagnosis and treatment of these benign neoplasms. 

## Case presentation

**Figure 1:**
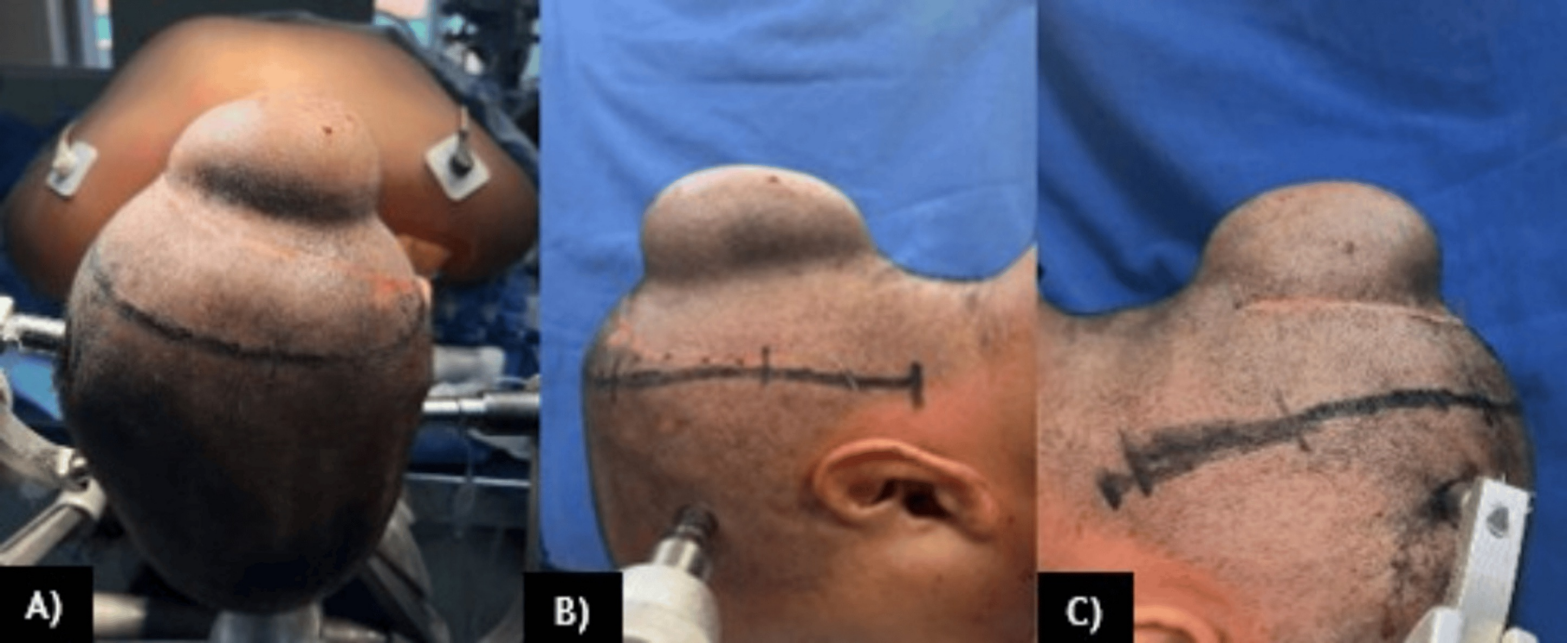
A-C) Painless mass in the occipital region of the skull of our patient prior surgery.

**Figure 2:**
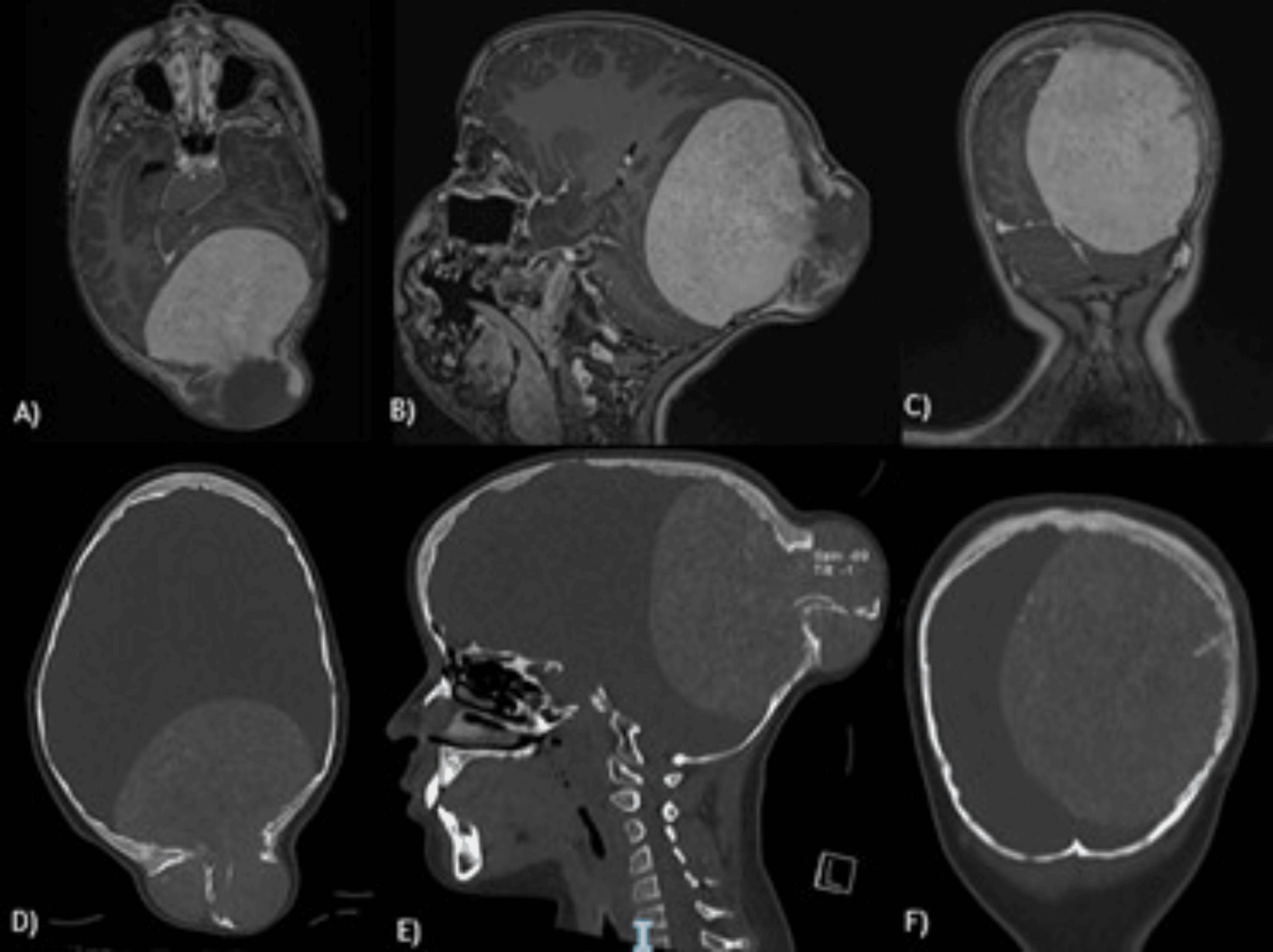
Axial, sagittal, and coronal T1 contrast sequences of the brain MRI and CT images. A) Axial, B) sagittal, and C) coronal T1 contrast sequences of the brain MRI show an extra-axial, heterogeneous, bilobed solid mass that largely enhances with gadolinium contrast in the occipital region of the skull. The mass compresses the brain tissue, especially the occipital lobe and cerebellar parenchyma, in the axial, sagittal, and coronal planes, respectively. Additionally, the sagittal superior sinus, torcula, straight sinus, and transverse sinus - especially the left one - are in contact with the tumor. D) Axial, E) sagittal, and F) coronal brain CT bone sequences show the tumor associated with destruction, hyperostosis, and reactive changes in the occipital bone. MRI, magnetic resonance imaging; CT, computed tomography

A nine-year-old Mexican girl with no significant medical history presented with a five-year history of a growing, painless mass in the occipital area of the skull, accompanied by headache. Rapid growth of the mass and progressive loss of visual acuity occurred in the last year, affecting her quality of life and school development (Figure 1). Because of the progression of symptoms, she was referred for evaluation. A biopsy was performed at another institution without conclusive results. Brain computed tomography (CT) and magnetic resonance imaging (MRI) revealed an extra-axial, bilobed lesion in the occipital bone that extends intra- and extracranially, with bone destruction, high vascularity, and extensive compression of the cerebral tissue (Figure 2).

**Figure 3:**
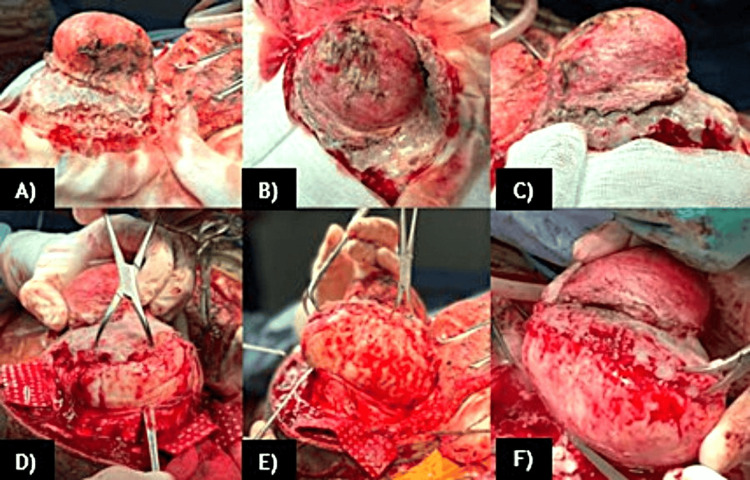
Surgical views of the lesion. A) Left view, B) superior view, and C) right view of the lesion. The dissection of the soft tissues and the exposure of the extracranial component of the lesion in relation to the occipital bone are shown. D-F) Sequential steps of the intracranial resection of the lesion, with adequate control of hemostasis and careful separation of adherent structures, such as the dura mater and venous sinuses adjacent to the tumor.

The patient scored 15 points on the Glasgow Coma Scale, with pupils at 3 mm, hyporeactive, and vision was finger counting at 1 m without papilledema. Motor and sensory functions were preserved. The patient underwent a horseshoe incision based on the cervical region; an occipital craniectomy was performed, and a complete en bloc tumor resection was carried out (Figure 3).

**Figure 4:**
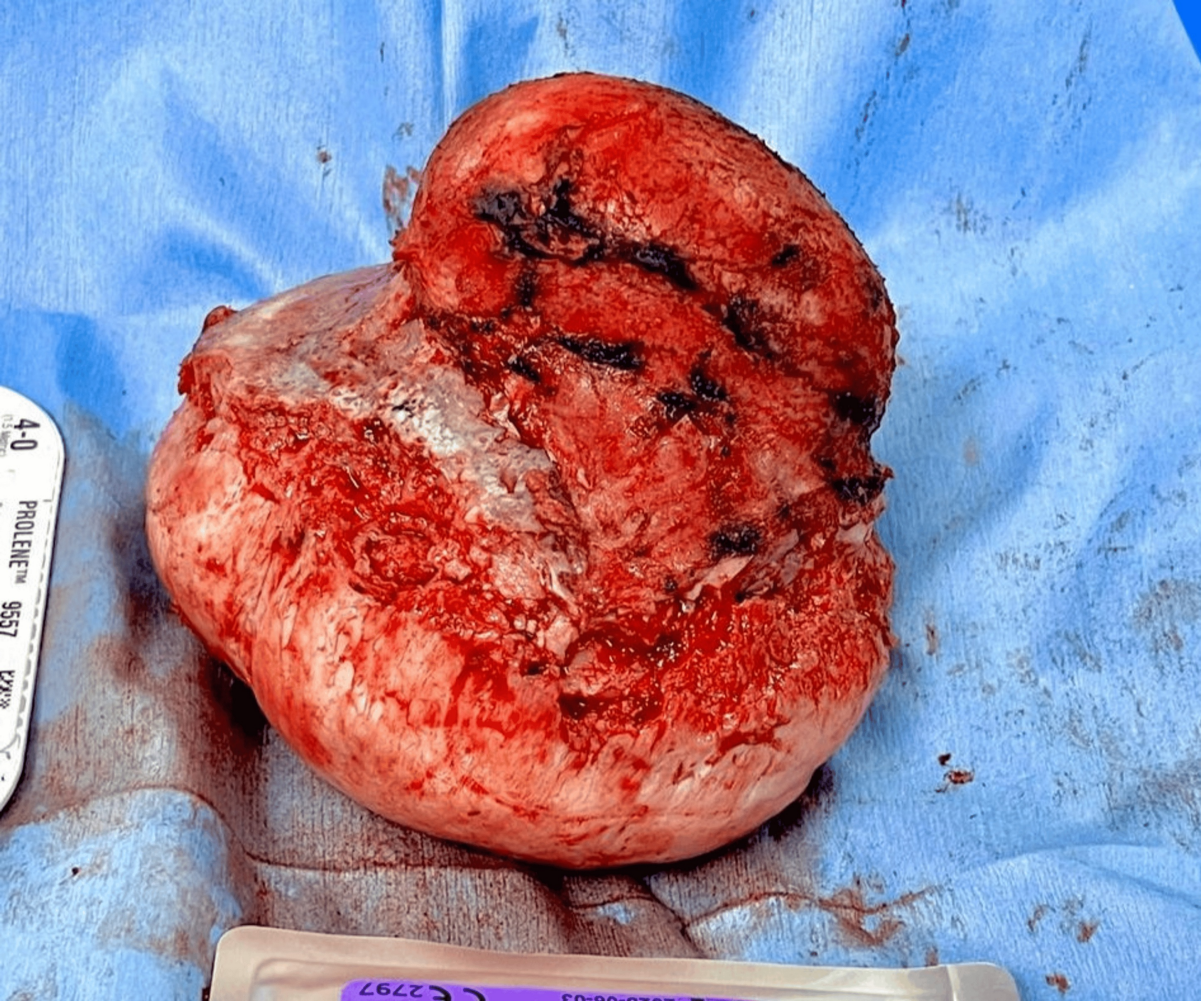
The bilobed lesion, measuring 11 × 14 × 10 cm, had a smooth, yellowish surface after total resection. The upper lobe of the lesion is the extracranial component and is shown in the upper portion of the image; on the other hand, the lower lobe corresponds to the intracranial component and is shown in the lower portion of the image.

The tumor was very hard in its composition and vascular, with evidence of a capsule. The surrounding bone tissue was very weak and friable to manipulation. The lesion was adhered to the dura mater and the external layers of the walls of the superior sagittal sinus, transverse sinuses, torcula, and straight sinus. Feeding vessels from the left occipital artery were identified and, with the use of bipolar cautery, were cut. Using Penfield dissectors with caution, we achieved total resection without damage to the venous sinuses. Two dural tears, each measuring 2 cm, were identified, and a primary duraplasty was performed. A bilobed lesion measuring 11 × 14 × 10 cm with a smooth, yellowish surface was removed, and between both lobes, a portion of flat bone measuring 11 × 9 cm was also excised. In serial sections, it was evident that the lesion was homogeneous, with a fibrous appearance that passed through a 5.6 cm defect in the bone tissue (Figure 4).

**Figure 5:**
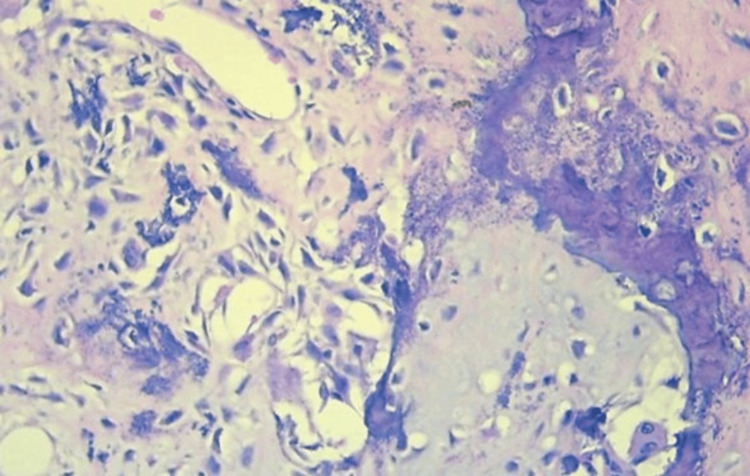
Microscopic findings in hematoxylin and eosin. Microscopic findings in hematoxylin and eosin stain show cell proliferation, consisting of spindle-shaped to polygonal cells with eosinophilic cytoplasm, without atypia, and deposits of hyaline matrix with an osteoid appearance and mineralization.

**Figure 6:**
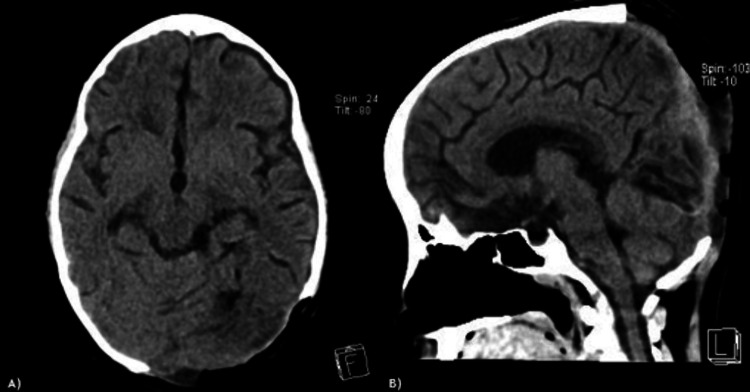
Brain CT images. A-B) Brain CT, 24 hours after surgery, shows complete resection without brain injury and expansion of the brain parenchyma. CT, computed tomography

Histopathological examination shows cell proliferation of mesenchymal origin, consisting of spindle-shaped to polygonal cells with moderately eosinophilic cytoplasm and ovoid nuclei with fine chromatin, without atypia. Between the cells, there is a deposit of dense hyaline matrix with an osteoid appearance and mineralization, all compatible with ossifying fibroma. Immunohistochemistry was not necessary due to the characteristic morphology under the microscope (Figure 5). Following surgery, the patient was extubated without complications, with a Glasgow Coma Scale score of 15, and experienced progressive improvement in vision, with no evidence of additional neurological deficits. The patient was discharged after one week, once the histopathological result was available. Postoperative imaging studies confirmed total resection with disease-free margins in the bone tissue and no injuries to the brain or venous structures (Figure 6).

## Discussion

To date, after a review of the literature on occipital ossifying fibromas, only five cases (including this one) have been reported. The first report, by Yamashita et al. in 1977 [4], described a nine-year-old girl without neurological deficit, presenting only with local swelling, who was treated by surgery without recurrence. In 1995, Binatli et al. [5] presented a case of a 13-year-old child with a painless occipital mass of one-year duration, measuring 5 cm in diameter. CT scans revealed a hyperdense lesion in the occipital bone restricted to the diploe, which was surgically removed en bloc without neurological deficit. Lam et al. [3] described a 49-year-old man who presented with progressive bilateral hand weakness and fifth-finger numbness over a two-year period. MRI showed an incidental extra-axial posterior fossa lesion measuring 4.7 × 3.1 × 3.6 cm, arising from the right occipital bone’s diploic space and causing destruction of both cortical tables. This mass showed enhancement on post-gadolinium images. The patient underwent right occipital craniectomy with complete excision of the tumor, which was confirmed as an ossifying fibroma [6]. Cotúa Quintero et al. [6] documented an 18-year-old male with a six-week history of headache and blurred vision. Examination revealed right VI nerve palsy, diplopia, and bilateral papilledema. Imaging confirmed a 6 × 4 cm left osteolytic parieto-occipital enhancing lesion causing a mass effect on the left cerebellum. Treatment included occipital artery embolization and total surgical removal of the lesion. The summary of the published cases is presented in Table 1. 

**Table 1:** Reported cases of occipital ossifying fibromas.

Case	Year	Gender	Age	Neurological deficit	Treatment	Symptoms	Subtype
Yamashita et al. [1]​​​	1977	Female	9	No	Surgery	Local swelling	Not described
Binatli et al. [5]	1995	Male	13	No	Surgery	Local swelling	Not described
Lam et al. [3]	2008	Male	49	Yes	Surgery	Progressive weakness of both hands	Not described
Cotúa Quintero et al. [6]	2016	Male	18	Yes	Surgery	Blurred vision, VI nerve palsy	Psammomatoid
Castruita Meza et al. (present study)	2025	Female	9	Yes	Surgery	Progressive loss of vision	Not described

Based on the cases reported, to our knowledge, our case is the largest in size. It is important to mention that the benign biological behavior of ossifying fibroma allowed en bloc resection, as usually no dural or brain tissue infiltration is found. While ossifying fibroma is a common benign lesion in craniofacial bones, its occurrence within the skull - specifically the occipital bone - is especially uncommon. Most cases (75%-89%) originate in the mandible, followed by the maxilla; the paranasal sinuses and orbit are less frequently involved [3].

While the frontal bone represents the most common site for calvarial lesions, these instances are typically linked to disease in the frontal, ethmoid, or sphenoid sinuses [3]. Ossifying fibromas affect young people; no sex predilection has been reported, and they usually manifest as painless swelling, occasionally growing rapidly and leading to intracranial hypertension [3]. Fibrous dysplasia represents a main differential diagnosis for ossifying fibromas. The key distinctions are that ossifying fibromas are typically monostotic and well-defined, lacking the ground-glass attenuation seen in fibrous dysplasia, which tends to be more elongated due to its developmental, non-neoplastic origin [6]. Some authors have even hypothesized that cranial trauma or radiation are risk factors for the development of this type of benign tumor, but this has not been confirmed [7]. Genetically, the absence of an activating mutation of the GNAS1 gene allows differentiation of ossifying fibromas from fibrous dysplasia [7]. Based on cases reported in the literature, no risk factors have been identified for the development of the neoplasm, and no recurrences have been reported. In all cases, complete resection was achieved, and four of the patients were young. To our knowledge, our case is only the second reported with a central neurological deficit associated with the location of the tumor. 

While radiological findings are helpful for diagnosis, they are challenging to interpret due to the lesion’s similarities with other craniofacial fibro-osseous lesions. On CT scans, the lesion may appear as a circumscribed, multiloculated, expansile mass with a thick bony wall, while post-contrast MRI might show enhancement of the lesion [8]. Optimal management involves complete surgical excision; failure to achieve total resection often results in recurrence rates documented between 30% and 56%. It is important to mention that malignant degeneration has not been reported [6]. Recurrence following partial removal of the lesion is a major concern; adjuvant treatment for these specific cases is not yet established. Thus, if recurrence occurs, clinical and radiological surveillance is typically the chosen approach [9]. An elevated risk of recurrence is associated with lesions exhibiting significant cortical destruction and periosteal elevation, mandating the achievement of tumor-free resection margins. When located in the cranium, open surgical management is favored, as it facilitates superior visibility and exposure of the mass and surrounding areas of potential extension [8]. We performed a complete excision of the lesion with macroscopic tumor-free margins due to the high risk of recurrence identified, such as cortical destruction. The patient has a good prognosis due to overall tumor remission and improvement in symptoms; cranioplasty for the skull defect is planned soon. 

## Conclusions

The benign biology of ossifying fibroma, and its rare invasion of the dura mater and brain tissue, allows - in experienced hands - total resection without additional neurological deficits, as in the case we present. The uncommon presentation of bone tumors like ossifying fibroma in the skull, with locally aggressive behavior, should always be considered in the differential diagnosis in the pediatric population, because total resection decreases morbidity and is curative. The limited availability of literature on the management and follow-up of this presentation limits recommendations; however, we advise safe, complete resection whenever possible, with adequate hemostasis control, as well as long-term imaging surveillance with MRI, as described for other benign lesions of the nervous system. 
